# Shared vision and autonomous motivation vs. financial incentives driving success in corporate acquisitions

**DOI:** 10.3389/fpsyg.2014.01466

**Published:** 2015-01-06

**Authors:** Byron C. Clayton

**Affiliations:** Case Western Reserve UniversityCleveland, OH, USA

**Keywords:** shared vision, mergers and acquisitions, self-determination theory, autonomous motivation, financial incentives, extra-role behaviors, M&A

## Abstract

Successful corporate acquisitions require its managers to achieve substantial performance improvements in order to sufficiently cover acquisition premiums, the expected return of debt and equity investors, and the additional resources needed to capture synergies and accelerate growth. Acquirers understand that achieving the performance improvements necessary to cover these costs and create value for investors will most likely require a significant effort from mergers and acquisitions (M&A) management teams. This understanding drives the common and longstanding practice of offering hefty performance incentive packages to key managers, assuming that financial incentives will induce in-role and extra-role behaviors that drive organizational change and growth. The present study debunks the assumptions of this common M&A practice, providing quantitative evidence that shared vision and autonomous motivation are far more effective drivers of managerial performance than financial incentives.

## Introduction

The poor financial returns and high failure rates of mergers and acquisitions (M&A) have been thoroughly documented. Researchers have indicated that approximately 70–80% of mergers and acquisitions do not create significant value above the annual cost of capital (Bruner, 2002). Even conservative estimates place M&A failure rates at approximately 50% or higher for nearly four decades (Kitching, 1974; Rostand, 1994; Coffey et al., 2003). Despite this conspicuously disappointing history, global M&A activity continues to increase at a phenomenal rate climbing from $1.9 trillion in 2004 (Cartwright and Schoenberg, 2006) to a record-breaking $4.35 trillion in 2007 (Reuters, 2008). With trillions of dollars in transactions at risk each year, it is extremely important for researchers and practitioners to find ways to curb M&A failures.

In order to succeed, M&As must create value for its investors despite new costs such as servicing debt, funding growth, and increasing return expectations to accommodate acquisition premiums^1^ (Sirower, 2000). These burdens require M&A managers to increase the performance of their firms to new heights. Acquirers typically offer substantial financial incentives to induce these managers to go above and beyond their normal job duties to champion aggressive organizational change and growth (Hitt et al., 2001).

This expectation is in direct conflict with social psychology theories such as Self-Determination Theory (SDT) (Gagne and Deci, 2005) and economic theories such as Motivation Crowding Theory (Frey and Jegen, 2001; James, 2005) which assert that financial incentives can reduce motivation and performance. Certainly, the persistently high M&A failure rates suggest the possibility that these theories apply to M&A managers.

The purpose of the present study is two-fold. First, it explores the effects of financial incentives on the motivation and performance of M&A managers. For completeness, our concept of managerial performance includes both in-role and championing behaviors. Second, the study explores two other practices that are used by acquirers to increase the performance of its acquired managers, increasing organizational support and focusing on shared vision.

### The need for innovative approaches to M&A research

Fifty years of M&A research have had no measurable impact on failure rates (Cartwright, 2005). Scholars continue to be bewildered by the conflicting and seemingly unpredictable performance of mergers and acquisitions (Tichy, 2001; King et al., 2004; Stahl and Voigt, 2004). M&A research has primarily focused on three streams of inquiry to identify the root cause(s) of failures: strategic fit, culture fit and integration process (Cartwright and Schoenberg, 2006). While these research paths have contributed much to our understanding of organizational-level changes related to M&A, neither has provided a consistent explanation of how and why these changes affect firm performance (King et al., 2004; Cartwright, 2005).

Scholars have offered several suppositions why existing M&A literature has not been effective. First, although psychological theorists consistently argue that human factors are the key to M&A success or failure (Cartwright and Cooper, 1996; Terry, 2003), 95% of existing M&A literature focuses on organizational-level constructs (Cartwright, 2005). This is rather surprising since organizational change, particularly the accelerated change experienced by most M&As, is usually if not always mediated through individual change (Schein, 1980; Schneider et al., 1996; Edmonson, 1999; Devos et al., 2001). M&A literature fails to predict the performance of merged or acquired companies simply because it cannot predict the performance of the managers charged with running these companies (Cartwright, 2005).

Two other suppositions suggest that most M&A studies (a) have not been theory-driven or (b) have been limited to case studies, both of which lack the generalizability to offer far-reaching solutions (Hogan and Overmeyer-Day, 1994; Seo and Hill, 2005). As a result, countless organizational practices have been prescribed for M&A planning and integration without a sound theoretical or empirical basis, certainly contributing to the high rate of failures (Seo and Hill, 2005). In fact, researchers concluded that “changes to both M&A theory and research may be needed” after analyzing the inability of 93 studies to clearly identify antecedents that consistently impact M&A performance (King et al., 2004). Evidently, unique approaches to M&A research is just as important as the research focus.

## Conceptual overview

In a ground-breaking analysis of M&A failures, Sirower (2000) illustrated how most M&A management teams face massive required performance improvements (RPIs) to achieve M&A success. The basic M&A premise involves purchasing a company at *X* price, then growing its value to *X* + *Y* at some designated time in the future. The value of Y must be sufficiently large enough to cover the acquisition premium, the expected return of debt and equity investors, surges in competitive activity responding to the M&A threat, additional resources requirements needed for growth and capturing synergies, acquisition transaction and consulting costs, executive contractual costs, and many other costs including the time value of money (Sirower, 2000). For M&A managers, status quo performance of in-role behaviors, no matter how efficient, will no longer suffice. Management performance must often substantially improve to meet the RPIs dictated by the need to achieve value *Y*. As such, M&A managers are expected to *champion* aggressive organizational change and growth to have any chance of achieving M&A success.

Understanding this dynamic, acquirers typically focus on three areas to improve in-role behaviors and more importantly, to induce championing behaviors from acquired managers: financial incentives, organizational support and shared vision. The most common and longstanding practice is to increase financial performance incentives via some combination of stock options, profit sharing, gain sharing or individual bonuses. In fact, acquirers often establish financial incentives as part of the transaction terms for key managers and immediately after the transaction for other managers (Hitt et al., 2001; Cullinan et al., 2004). Acquirers understand that any delays in achieving performance improvements quickly compound the returns needed to accommodate value *Y*. Sirower (2000) calculated that expectations of a 10% return on equity (ROE) would increase to approximately 15% on minimal or substandard returns for the first couple of years after an acquisition. This 50% increase in ROE would have to be maintained for the following 7 years just to break even. In other words, his analysis assumes no value creation, only value preservation for the acquirer. Consequently, acquirers typically provide substantial performance incentives expecting they will induce key managers to champion whatever changes are necessary to achieve the acquirer's goals. This practice is supported by empirical studies on compensation, which in general, report a positive influence of monetary incentives on employee and firm performance (Booth and Frank, 1999; Lazear Edward, 2000; Gerhart and Rynes, 2003; Gagne and Forest, 2008).

Unfortunately, the exceptionally high rates of M&A failures indicate that increasing performance incentives do not consistently increase the performance of acquired managers. This directly contradicts common practice and general compensation literature. However, economics and social psychology scholars have provided theory and corresponding empirical evidence describing certain conditions where financial incentives are ineffective and in fact, can actually undermine motivation and performance (Gagne and Deci, 2005; James, 2005). This suggests the presence of a mediator that suppresses the total effect of financial incentives on performance.

This mediation effect is supported by SDT (Gagne and Deci, 2005). SDT posits that individuals perceive financial incentives as control mechanisms. As such, financial incentives reduce individuals' autonomous motivation, that is, their willingness to act on organizational goals according to their own volition.

**Figure 1:**
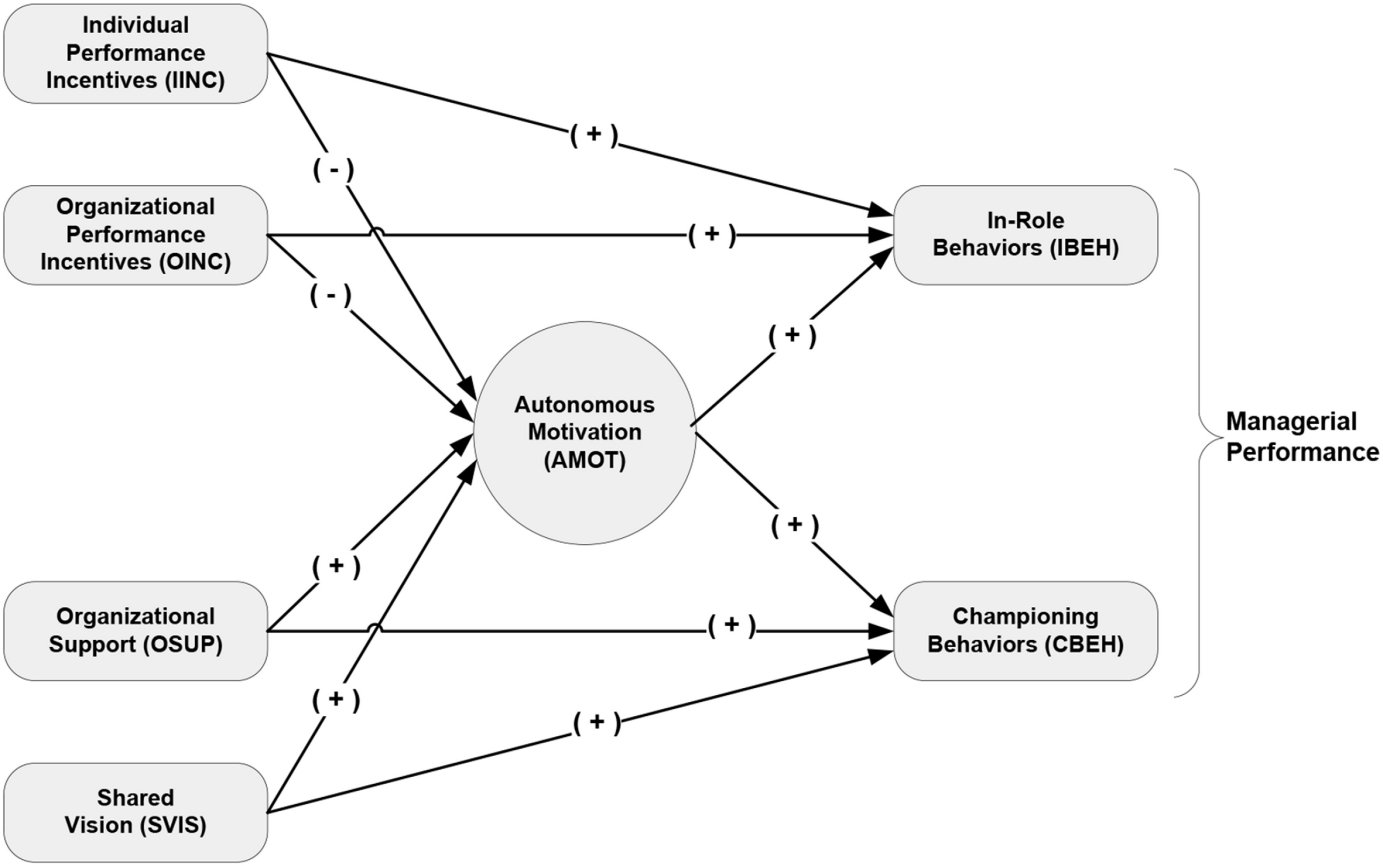
**Conceptual model**.

Figure 1 graphically depicts the aforementioned assertions regarding the direct and mediation effects of financial incentives on in-role and championing behaviors in a single model. The model illustrates the positive relationship between incentives and performance behaviors espoused by general compensation literature and M&A practice. It also illustrates the negative mediation effect of autonomous motivation espoused by SDT.

Providing organizational support is another common method acquirers use to improve in-role performance and induce championing behaviors. When parent organizations actively demonstrate a concern for acquired employees' well-being, threat is reduced, motivating employees to willingly reciprocate with actions that contribute to the well-being of the organization (Gaertner et al., 2001; Seo and Hill, 2005). Two of the most successful and studied serial acquirers, Cisco Systems and GE Capital, both consider their organizational support of acquired employees during post-acquisition integration as the key to their success (DiGeorgio, 2001). Figure 1 depicts organizational support as positively impacting both autonomous motivation and managerial performance.

Shared vision is the third common method used by M&A practitioners to maximize performance (Douma et al., 2000; Mitleton-Kelly, 2004; Stahl and Mendenhall, 2005). We define shared vision as a manager's focus and alignment toward the new regime's direction and purpose. M&A literature asserts that a shared vision is essential to the successful performance of merged and acquired organizations (Haspeslagh and Jemison, 1991; Sitkin and Pablo, 2005). J. P. Garnier, the former CEO of GlaxoSmithKline, extensively discussed the importance of management's focus on shared vision when analyzing GSK's many M&A successes (Stahl and Mendenhall, 2005). Cisco System explores shared vision as part of the pre-acquisition process while GE Capital requires its integration managers to develop shared vision during the post-acquisition integration process as part of their role (DiGeorgio, 2001). Per Figure 1, we posit that shared vision positively influences both autonomous motivation and championing behaviors.

### Managerial performance

Our definition of managerial performance consists of in-role behaviors and championing behaviors. In-role behaviors are defined and required by formal job descriptions (Williams and Anderson, 1991; Riketta, 2002). They are recognized and driven by an organization's formal reward system (Barksdale and Werner, 2001). Researchers most often characterize in-role behaviors as simply “doing one's job.”

Championing behaviors are a form of extra-role behaviors based on the “taking charge” construct defined by Morrison and Phelps (1999). Like other forms of extra-role behaviors, they are discretionary actions that are not defined or enforced by formal role obligations (Morrison and Phelps, 1999). Unlike other forms of extra-role behaviors, they are specifically change-oriented, describing individuals who are willing to challenge the status quo to bring about constructive organizational change (Morrison and Phelps, 1999). Championing behaviors describe voluntary efforts to continuously improve organizational functioning. Researchers have statistically confirmed their relation to but distinction from other forms of extra-role behaviors (Morrison and Phelps, 1999; Chiaburu and Baker, 2006).

### The direct effects of financial incentives on in-role performance

Performance incentives for acquired managers can take several forms. Table 1 lists the most common incentives categorized by their basis of evaluation. Researchers over the past decade have consistently reported that at least 95% of U.S. companies provide performance incentives with approximately 35% of those companies providing individual-based incentives and 60% providing organizational-based incentives (Bucklin and Dickinson, 2001; McGee et al., 2006). Because increasing performance is so essential to the success of mergers and acquisitions (Sirower, 2000), we suspect that an even higher percentage of M&As provide some form of performance incentives.

**Table 1:** **Common performance incentives for managers**.

**Evaluation basis**	**Performance incentive**	**Description**	**Major pros and cons**
Individual performance	Individual performance bonus	Cash compensation based on achieving individual goals. Funded by pre-determined budget set aside for bonuses	Pros: Excellent influence on individual performance. Cons: Promotes self-interest and competition among peers
Organizational performance	Profit sharing bonus	Cash or deferred compensation based on the economic performance of the firm. Funded by firm profits	Pros: Signals willingness to share wealth with workforce. Easy to administer. Cons: Weak influence on day to day individual performance
Organizational performance	Gainsharing bonus	Cash compensation based on specific short or long-term operational goals. Funded by cost savings, increased revenue or productivity gains	Pros: Good to excellent influence on individual performance depending on the size of the group. Promotes cooperation, team work and positive peer pressure. Cons: Can be difficult to administer and keep current
Organizational performance	Stock or stock options	Stock or the right to purchase stock at a fixed price. Funded by the sale of the firm or of the firm's stock	Pros: Easy to administer. Can be a substantial amount. Cons: Weak influence on day to day individual performance

This common practice of using performance incentives is based on research on compensation showing financial incentives have a positive effect on employee performance (Gerhart and Rynes, 2003) with studies showing a 4–9% increase in firm performance (Booth and Frank, 1999; Lazear Edward, 2000; Gagne and Forest, 2008). Because financial strategists dominate M&A literature and practice (Sudarsanam, 2003; Cartwright, 2005), it is not surprising that they have adopted the perspective supported by the financial literature regarding compensation. M&A literature specifically recommends performance incentives that range from “cash compensation for particular actions to stock options and equity ownership” (Hitt et al., 2001), to drive “stimulating sustained, vigorous performance” (Larsson and Finkelstein, 2002), because “incentives matter a great deal in determining the success of an acquisition (Kaplan, 2000).

From a theoretical perspective, the direct effect of incentives on performance is based on the economic exchange model (Blau, 1964) that promises specific benefits from the organization in return for specific contributions from employees (Tsui et al., 1997). Equity Theory, one of many theories that utilize the economic exchange model, asserts that employees strive to balance the contributions they provide relative to the benefits they receive (Adams, 1965; Cropanzano et al., 2007). According to Equity Theory, financial incentives should positively influence the performance of an individual's specified behaviors, that is, in-role behaviors. Accordingly, studies have supported a positive correlation between performance incentives and in-role behaviors (Deckop et al., 1999), specifically referring to it as the pay-performance link (Bucklin and Dickinson, 2001).

*Hypothesis 1: Individual performance incentives positively influence in-role behaviors after controlling for autonomous motivation*.

*Hypothesis 2: Organizational performance incentives positively influence in-role behaviors after controlling for autonomous motivation*.

Compensation specialists have historically cited the pay-performance link to be the most important factor in determining the influence that financial incentives have on an individual's performance (Bucklin and Dickinson, 2001). The strength of the pay-performance link depends on the amount of control an individual has over achieving the targeted goals (McGee et al., 2006). The more control one has over achieving the goals, the more control he has over his pay.

One has much more control over achieving individual-based goals than group-based goals (McGee et al., 2006). Simply put, the less people involved in achieving a goal, the more that a single participant can control the outcome. In addition, organizational performance incentives are based on the impact of operational or economic outcomes on the firm, many of which depend on external variables that are clearly beyond the control of management (Bucklin and Dickinson, 2001) such as the economy, government regulation, customer demand, and competitor strategies. Even controllable variables such as productivity and quality are an aggregate based on the performance of all employees, meaning that an individual may perceive his contribution as insignificant toward achieving the incentive (FitzRoy and Kraft, 1995; Hall and Murphy, 2003). As a result, individuals may have difficulty seeing the connection between group-based incentives and their day-to-day performance of in-role behaviors (Bucklin and Dickinson, 2001; Hall and Murphy, 2003).

*Hypothesis 3: Individual performance incentives have a stronger (more positive) influence on in-role behaviors than organizational performance incentives*.

### The mediating effects of autonomous motivation on financial incentives

Mediating effects on financial incentives are not considered by the economic exchange based theories that dominate compensation research and practice (Frey and Osterloh, 2005; Gagne and Forest, 2008). However, numerous studies have explored how certain conditions can cause tangible incentives to undermine motivation and performance (Gagne and Deci, 2005; James, 2005). This “hidden cost of reward” was first identified and researched by social psychologists as far back as 1971 (Titmuss, 1971; Lepper and Greene, 1978; Frey and Oberholzer-Gee, 1997). Over the years, this concept has been included in many theoretical approaches to work motivation and performance such as Cognitive Evaluation Theory (Deci and Ryan, 1985) and Motivation Crowding Theory (Frey and Jegen, 2001; James, 2005).

**Figure 2:**
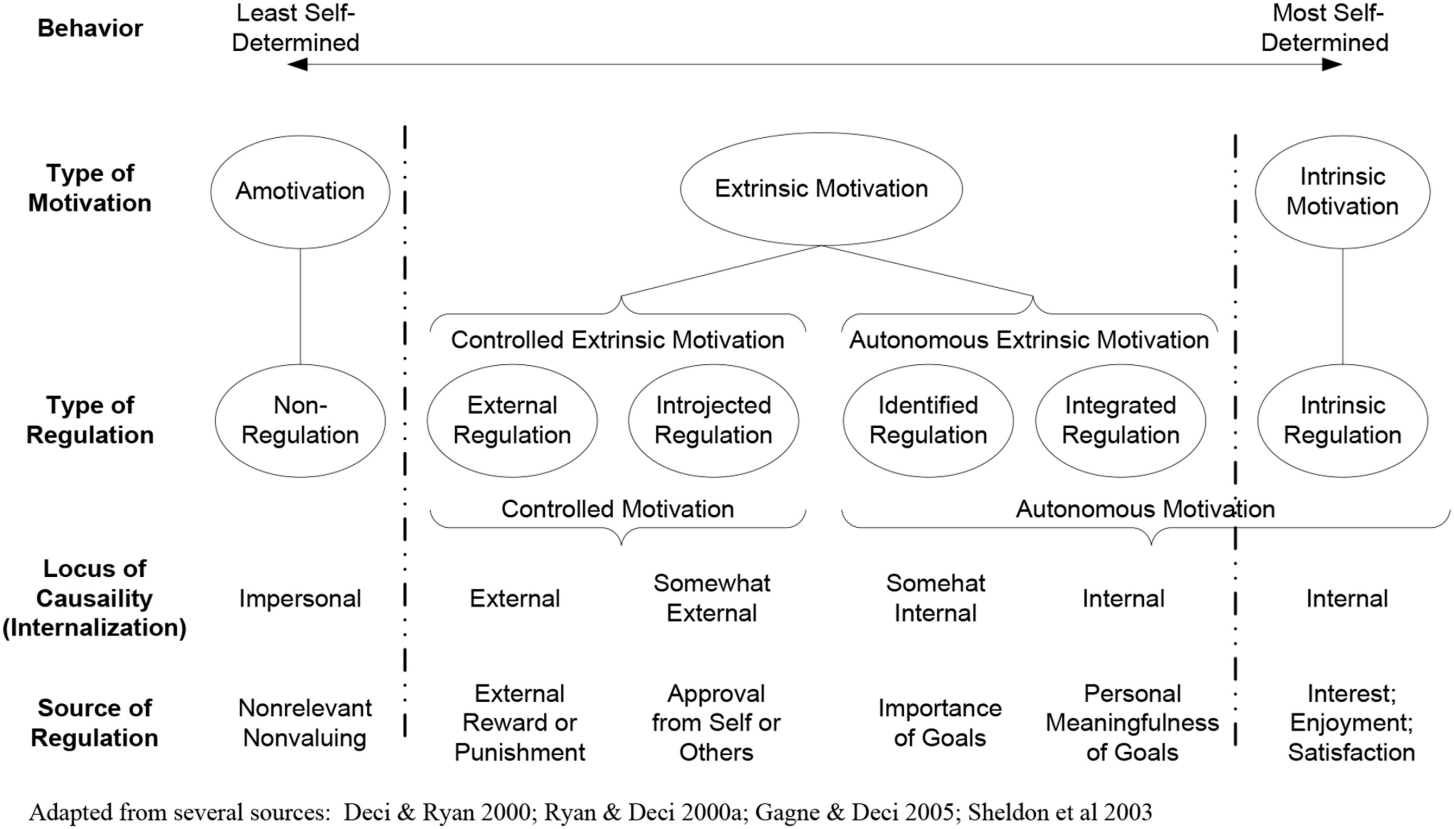
**Self-determination continuum**.

SDT posits that the perceptions of tangible incentives *regulate* an individual's motivation and behaviors (Deci and Ryan, 2000; Ryan and Deci, 2000a; Sheldon et al., 2003; Gagne and Deci, 2005). Referring to Figure 2, the Self-Determination Continuum, the *source of regulation* describes the reasons that individuals act on the organizational goals. When an individual generally regards organizational goals as personally important, meaningful or interesting, they are *internalized* or internally valued by the individual (Ryan and Connell, 1989; Sheldon et al., 2003; Gagne and Forest, 2008). High degrees of internalization enhance an individual's perceived autonomy, resulting in their willing intention to act on organizational goals (Deci and Ryan, 1987; Gagne and Deci, 2005). SDT defines this willing intention to act as *autonomous motivation*. Conversely, an individual that generally regards organizational goals with low degrees of internalization will only act on them to receive a reward, avoid a punishment or achieve the approval of others (Ryan and Deci, 2000a; Meyer et al., 2004). SDT refers to this as *controlled motivation*.

Figure 2 depicts the sources of regulation that include external rewards increase controlled motivation while reducing autonomous motivation. SDT states that individuals perceive tangible rewards as control mechanisms, attempting to force or coerce them into acting on organizational goals. SDT posits that tangible rewards reduce autonomous motivation.

*Hypothesis 4: Individual performance incentives negatively influence autonomous motivation*.

*Hypothesis 5: Organizational performance incentives negatively influence autonomous motivation*.

Researchers have proposed that organizational commitment is actually a component of work motivation (Meyer and Herscovitch, 2001; Meyer et al., 2004). Empirical research has confirmed considerable overlap between both major conceptualizations of organizational commitment (O'Reilly and Chatman, 1986; Meyer and Allen, 1991) and the SDT framework of work motivation (Gagne and Deci, 2005; Gagné et al., 2009). Affective organizational commitment, characterized as the willing desire to identify with an organization was specifically linked to autonomous motivation (Gagné and Koestner, 2002; Gagné et al., 2004; Gagne and Deci, 2005).

A number of studies have linked affective organizational commitment to in-role and extra-role behaviors. Researchers have established a significant overlap between affective organizational commitment and autonomous motivation. In a meta-analysis of 93 published studies, affective organizational commitment was found to (a) positively influence in-role behaviors, (b) to positively influence extra-role behaviors, and (c) to influence extra-role behaviors significantly more than in-role behaviors (Riketta, 2002). Similar results were also reported in a separate meta-analysis of 155 independent samples involving more than 50,000 employees (Meyer et al., 2002).

*Hypothesis 6: Autonomous motivation positively influences in-role behaviors*.

*Hypothesis 7: Autonomous motivation positively influences championing behaviors*.

*Hypothesis 8: Autonomous motivation has a stronger (more positive) influence on championing behaviors than in-role behaviors*.

Hypotheses 1 through 8 establish autonomous motivation as a mediator of the influence that performance incentives have on in-role and championing behaviors. Because SDT posits a negative relationship between financial incentives and autonomous motivation, our hypothesized model conceptualizes that autonomous motivation negatively mediates the impact of incentives on managerial performance. This is in accordance with SDT but directly conflicts with M&A practice.

*Hypothesis 9: Autonomous motivation negatively mediates the influence of individual performance incentives on in-role behaviors*.

*Hypothesis 10: Autonomous motivation negatively mediates the influence of organizational performance incentives on in-role behaviors*.

*Hypothesis 11: Autonomous motivation negatively mediates the influence of individual performance incentives on championing behaviors*.

*Hypothesis 12: Autonomous motivation negatively mediates the influence of organizational performance incentives on championing behaviors*.

### The effects of organizational support on managerial performance

The present study conceptualizes organizational support through the perceptions of each individual manager. Perceived organizational support (POS) captures an individual's beliefs concerning the degree to which an organization values their contributions and cares about their well-being (Eisenberger et al., 1986). POS develops from an employee's personification of the organization and its intent toward favorable or unfavorable treatment and working conditions (Rhoades and Eisenberger, 2002). High levels of POS indicate a work environment that is likely to exhibit fair treatment, participation in decision-making, career development and training, job security, recognition, supervisor support and a strong sense of belonging (Wayne et al., 1997; Rhoades and Eisenberger, 2002; Masterson and Stamper, 2003).

Social Exchange Theory (SET), a foundation for understanding the relationships between individuals and their organizations, posits that an individual is likely to reciprocate the favorable or unfavorable treatment received from an organization (Blau, 1964). On the basis of SET, POS should drive an individual's willing desire to act on behalf of the organization (Rhoades and Eisenberger, 2002). In a meta-analysis of over 70 studies on the antecedents and outcomes of POS, Rhoades and Eisenberger (2002) identified a strong and consistent positive relationship between POS and affective commitment. Given the established relationship between affective organizational commitment and autonomous motivation, we propose the following:

*Hypothesis 13: Organizational support positively influences autonomous motivation*.

Rhoades and Eisenberger's meta-analysis (2002) also reports that extra-role behaviors toward the organization as a significant outcome of POS. A recent study corroborates those findings, specifically confirming that POS is the antecedent to extra-role behaviors (Chen et al., 2009).

*Hypothesis 14: Organizational support positively influences championing behaviors*.

Hypotheses 6, 7, 13, and 14 establish autonomous motivation as a mediator of the influence that organizational support has on in-role and championing behaviors. The positive effects conceptualized for each hypothesis posits that autonomous motivation positively mediates the impact of organizational support on managerial performance.

*Hypothesis 15: Autonomous motivation positively mediates the influence of organizational support on in-role behaviors*.

*Hypothesis 16: Autonomous motivation positively mediates the influence of organizational support on championing behaviors*.

### The effects of shared vision on managerial performance

M&A literature asserts that a shared vision, defined as a common direction and purpose among an M&A's leaders and employees, is essential to its successful performance (Haspeslagh and Jemison, 1991; Sitkin and Pablo, 2005). A shared vision of an organization's future must be consistently encouraged and communicated to take root, spread and foster an environment of excellence (Senge, 1990). An effective shared vision provides the focus, direction and purpose for day-to-day individual efforts. They remind us of the meaning and importance of our work (Boyatzis and McKee, 2005).

Shared vision is particularly important in M&A environments because it is a bonding mechanism that helps different parts of an organization combine resources which promotes the integration of the entire organization (Tsai and Ghoshal, 1998). At an individual level, shared vision creates an emotional bond between an employee and his organization, providing a common identity and sense of belonging (Senge, 1990; Dvir et al., 2004). This sense of belonging enhances relatedness, which according to SDT, increases autonomous motivation In other words, an individual who agrees with or is inspired by the organization's vision is more likely to willingly act on behalf of the organization (Ashforth and Humphrey, 1995; Dvir et al., 2004).

*Hypothesis 17: Shared vision positively influences autonomous motivation*.

Intentional Change Theory posits that shared vision drives the types of behaviors that cultivate and sustain individual, group and organizational change (Akrivou et al., 2006; Boyatzis, 2006; Van-Oosten, 2006). We contend that these behaviors are closely related, if not identical to championing behaviors. Championing behaviors consist of discretionary conduct focused on implementing constructive organizational change (Morrison and Phelps, 1999; Chiaburu and Baker, 2006).

*Hypothesis 18: Shared vision positively influences championing behaviors*.

Hypotheses 6, 7, 17, and 18 establish autonomous motivation as a mediator of the influence that shared vision has on in-role and championing behaviors. The positive effects conceptualized for each hypothesis posit that autonomous motivation positively mediates the impact of shared vision on managerial performance.

*Hypothesis 19: Autonomous motivation positively mediates the influence of shared vision on in-role behaviors*.

*Hypothesis 20: Autonomous motivation positively mediates the influence of shared vision on championing behaviors*.

## Research methods

### Sample

The study focuses on mergers and acquisitions owned by private equity firms. Private equity firms typically have a 5–7 year turnaround timetable for their investments (Flanigan, 2005). To this end, managers must implement aggressive, short-term growth strategies designed to quickly improve the firm's performance to levels never before achieved. The study targets firms that were acquired at least 3 months prior to the survey. M&As less than 3 months old have not had sufficient time for changes in financial incentives to have an effect on the attitudes and behaviors of acquired managers. Three months is a common milestone used by M&A practitioners to judge the direction of early-stage change implementation (DiGeorgio, 2001; Bertoncelj and Kovac, 2007).

Companies owned by private equity provide an excellent climate to evaluate the drivers of managerial performance. Private equity ownership fosters an environment of time-constrained, aggressive-growth expectations that require high levels of in-role and championing behaviors from M&A managers.

The sample consists of CEOs, senior managers and middle managers from 54 M&As owned by a large private equity firm headquartered in North America The M&As are middle market companies with revenues ranging from $5–500 million annually that compete in a variety of industries. The study defines senior managers as those who report directly to the CEO or president and middle managers as one or two reporting levels below senior managers. The sample provides a comprehensive representation of managers that receive individual and organizational based performance incentives. CEOs and senior manages usually participate in incentive plans based on stock performance, receiving stock or stock options. Middle managers usually participate in incentive plans based on organizational performance such as gainsharing or profit sharing. Both senior and middle managers commonly receive incentives based on individual performance in addition to stock or organizational incentives. Table 2 provides a description of the respondents who completed surveys.

**Table 2:** **Description of respondents**.

**Management level (MLVL)**			**Acquisition age (AAGE)**			**Age (PAGE)**			**Tenure (PTEN)**			**Gender (PGEN**		
CEOs	23	8%	90 days—3 years	120	39%	18–29	68	22%	<3 years	98	32%	Male	254	83%
Senior managers	90	29%	3+ year	186	61%	30–44	154	50%	3–10 years	96	31%	Female	52	17%
Middle managers	191	62%				45+	84	27%	10+ years	112	37%			
Non-managers	2	1%												

### Data collection

The researchers developed an online survey designed to collect self-report data regarding performance incentives, motivation, shared vision and organizational support. A secondary survey and procedure were designed to collect performance data from each respondent's immediate supervisor. Unfortunately, the sponsoring organization did not approve the secondary survey for distribution to its managers. Therefore, the researchers expanded the primary survey to include self-reported performance. IRB exemption was obtained but all protocols governing use of human subjectgs were followed. Out of 500 managers solicited, 306 returned completed surveys for a 61% response rate.

#### Measures

Each of the measures used to develop the survey were based on existing validated scales using 5 point Likert responses with the exception of the performance incentive measures and controls. The performance incentive items simply reported the level of financial inducements as a percentage of base salary. The controls were reported management level or demographic information.

#### Performance incentives

Individual performance incentives are financial bonuses based solely on the performance of the manager in relation to formal job duties. Organizational performance incentives are financial rewards based on the performance of the organization. Organizational incentives include profit sharing bonuses, gainsharing bonuses and stock options. Being a report of factual data, these items followed the standard practice of measuring financial incentives as percentages or multiples of base salary (Murphy et al., 1999).

#### Autonomous motivation

The items chosen to measure autonomous motivation were adapted from the Relative Autonomy Index (RAI) originally developed by Ryan and Connell (1989) and its subsequent adaptations (Williams and Deci, 1996; Black and Deci, 2000). The Relative Autonomy Index measured each type of motivation described by SDT according to its degree of autonomy (Millette and Gagné, 2008). The RAI is computed by subtracting the scores from its controlled motivation subscale from its autonomous motivation subscale, such that the more positive scores indicate higher levels of autonomous motivation (Deci and Ryan, 2008). However, according to Deci and Ryan (2008), analyses can also be conducted using either of the two subscales. Three items for each subscale were selected for this study—the item numbers correspond to the item number in the original scales.

#### Organizational support

The researchers chose five items from the nine-item version of the POS scale (Eisenberger et al., 1990; Wayne et al., 1997) to operationalize organizational support. The POS scale describes employee perceptions about the extent an organization values their contributions and cares about their well-being. The items were selected because of their more consistent Cronbach's alpha coefficients of 0.81–0.93 as compared to the original (36-item) and revised (17-item) versions with reliabilities ranging from.074 to.095 (Wayne et al., 1997; Moorman et al., 1998; Fields, 2002). The five items selected for this study have the item numbers corresponding to the item number in the original scale.

#### Shared vision

Five items were selected from the vision subscale of the PNEA Survey (Boyatzis, 2008) to measure shared vision. The PNEA vision subscale measures the respondent's focus on and alignment with the organization's vision. The five items were chosen (from eight items in the referenced subscale) because of their particular relevance to the context of the present study. The five items selected for this study have the item numbers corresponding to the item number in the original scale.

#### In-role behaviors

Managerial in-role behaviors were assessed using five items adapted from a multi-dimensional scale designed to measure employee performance in the workplace (Williams and Anderson, 1991; Turnley et al., 2003). The in-role behaviors subscale specifically measures behaviors recognized by the formal reward system (Williams and Anderson, 1991; Turnley et al., 2003). This measure has been extensively used for peer, supervisor and self-reports (Fields, 2002).

#### Championing behaviors

Championing behaviors were measured by the “taking charge” scale which was developed to assess an individual's discretionary actions toward organizational change (Morrison and Phelps, 1999). These types of extra-role behaviors challenge the status quo by implementing changes or correcting problems in an effort to constructively improve organizational functioning. Championing behaviors are distinctively different from the altruistic, conscientious or civic virtue behaviors measured by most extra-role or organizational citizenship instruments and therefore, require a specific assessment tool (Morrison and Phelps, 1999; Chiaburu and Baker, 2006). The researchers chose the taking charge scale to operationalize championing behaviors because it targets the aggressive, change-oriented behaviors most M&As require from their managers to succeed. Five items were adapted to weigh the respondent's efforts toward solving pressing organizational problems or implementing new systems, technologies or methodologies, all of which are essential for accelerating M&A growth and performance.

#### Control variables

The influence of incentives, organizational support and shared vision may also vary with managerial hierarchy. Higher-level managers are likely to have different informational and interpersonal relationships with parent organizations, which could result in different attitudes (Tsui et al., 1997). A multigroup analysis was conducted to evaluate structural model invariance across senior managers and middle managers. The process involves comparing the goodness of fit between a model with structural paths constrained equal across groups to a model with no constraints (Byrne, 2001). A significant difference in chi-square indicates the models are not invariant, warranting each constraint to be released, one at a time, to pinpoint the specific paths causing the variance.

Multigroup analysis was also used to assess model invariance of managers under 45 years old to those 45 and older. Part of the M&A due diligence effort, when considering an acquisition, is to evaluate the management team. While some acquirers prefer younger management teams, feeling they are more flexible and dynamic, other acquirers prefer older management teams, feeling they are more experienced and knowledgeable (Wiersema and Bantel, 1992). The multigroup analyses was undertaken to identify if performance incentives, organizational support and shared vision affected the behaviors of senior and middle managers differently.

Other factors may impact M&A managers as well. Gender and company tenure are often considered as human capital factors that influence workplace performance (Tsui et al., 1997). As such, we included these items as control variables.

### Method of analysis

Normality, homoscedasticity and multicollinearity were examined; extreme outliers and influentials removed, and linear relationships were confirmed. An exploratory factor analysis (EFA) followed to uncover the latent structure of the measurement model in relation to a priori assumptions. The resulting measurement model was then subjected to a confirmatory factor analysis (CFA) to assess its fit to the data using structural equation modeling (SEM) methodologies. The researchers used SPSS and AMOS statistical software packages to conduct data and measurement model analyses.

Common method variance was of particular concern in the present study because the survey instrument was administered at the same time, in the same context, to single respondents, all of which can contribute to inflating the relationships between constructs (Podsakoff et al., 2003; Friedrich et al., 2009). Podsakoff et al. (2003) advocated the single-common-method-factor approach to control for CMV, particularly when the predictor and criterion measures were obtained from the same source and in the same context, as in this case. The procedure calls for establishing a latent factor in the measurement model which loads on each observed item. The main advantage of this approach is that it does not require the researcher to identify and measure specific causes of CMV. Unfortunately however, this approach also reflects the variance for other unmeasured variables in addition to CMV (Podsakoff et al., 2003). Other disadvantages include the tendency for this approach to result in under-identified models, particularly when the number of items is small in relation to the number of constructs, as in this case. As a solution to this problem, some researchers constrain the CMV factor loadings to be equal (Podsakoff et al., 2003). We referenced this approach to control for CMV during the analyses of measurement and structural models.

Prior to analyzing the hypothesized structural model, convergent and discriminant validity of the constructs as well as their internal reliability were assessed and confirmed. SEM techniques were then used to evaluate the causal relationships between the constructs in the structural model (Fornell and Larcker, 1981; Byrne, 2001). Goodness of fit statistics included measures comparing predicted vs. observed covariances (chi-square, relative chi-square, and SRMR), default vs. independence models (CFI, NFI, and TLI), and predicted vs. observed covariances penalized for lack of parsimony (RMSEA). To summarize the causal relationships between constructs, the *r*^2^ statistics for each mediating and dependent variable were tabulated with the unstandardized regression coefficient and *t*-value for each of its contributing explanatory variables.

Mediation testing followed the approach advocated by Mathieu and Taylor (2006). This approach incorporates iterative, systematic techniques designed to test for partial mediation, full mediation and indirect effects models. Its primary focus is on identifying indirect effects, specifically those that may suppress the total effects between predictor and criterion variables, causing many researchers to overlook important mediating relationships. The a priori assumptions of suppression effects in our hypothesized model warranted the use of this approach.

Finally, we examined the effects of the designated controls on our results. Management level and participant age were of particular interest. Therefore, multigroup analysis procedures were used to test the invariance of our model across senior and middle managers, as well as managers under and over 45 years old. Both measurement and structural models were tested for invariance.

## Results

SEM requires sample sizes greater than 200 with five to ten cases per observed variable (Kline, 2005; Hair et al., 2006). The original dataset consisted of 306 cases and 28 observed variables, meeting the data adequacy requirements for SEM. Subsequent analyses resulted in a final dataset of 285 cases and 20 observed variables, still exceeding the minimum requirements for SEM.

The pre-screening process identified two cases of respondents who were not CEOs, senior managers or middle managers. The cases were removed from the dataset. SPSS generated box plots, stem and leaf diagrams, and histograms as well as skewness and kurtosis values were examined to confirm acceptable normality of the observed variables. The analysis resulted in the identification and removal of 11 outliers from the dataset.

Each dependent variable was regressed on all independent variables which confirmed linear relationships suitable for SEM analysis. For each regression, SPSS generated plots of the standardized residuals against the standardized predicted value to confirm homoscedasticity of the variables. Finally, the regressions also produced collinearity statistics, confirming that all tolerance and VIF statistics were below the acceptable multicollinearity thresholds of <0.10 and >10, respectively (Kline, 2005).

An EFA was conducted using Principal Axis Factoring and Promax rotation to uncover the minimum number of factors required to account for the maximum amount of common variance assuming oblique relationships, not orthogonal. Twenty-eight observed variables were loaded into SPSS for EFA analysis. The items measuring individual and organizational performance incentives were not included in EFA. These measures assessed the amount and type of incentives reported by the respondents. As such, they were single item measures that did not indicate latent variables. The initial EFA resulted in a five factor solution with most variables loading as hypothesized. However, several of the items had cross-loadings within 0.200, had factor loadings below 0.500, or would improve the Cronbach's alpha of the construct if it were deleted. After several iterations, a total of 10 items were removed, resulting in a clean five factor solution as depicted in Table 3.

**Table 3:** **Simultaneous EFA of observed variables with rotated factor loadings (n-285, EFA conducted with principal axis factoring and promax rotation and kaisaer normalization)**.

**Variables**	**Factor 1**	**Factor 2**	**Factor 3**	**Factor 4**	**Factor 5**
**AUTONOMOUS MOTIVATION (AMOT)**					
AREG1					0.620
AREG3					0.896
AREG 6					0.833
**ORGANIZATIONAL SUPPORT (OSUP)**					
OSUP1				0.588	
OSUP4r				0.898	
OSUP5				0.950	
**SHARED VISION (SVIS)**					
SVIS1		0.952			
SVIS2		0.919			
SVIS3		0.842			
**IN-RLE BEHAVIORS (IBEH)**					
IBEH1	0.962				
IBEH3	0.858				
IBEH5	0.916				
**CHAMPIONING BEHAVIORS (CBEH)**					
CBEH1			0.522		
CBEH2			0.505		
CBEH3			0.773		
CBEH4			1.047		

Referring to Table 3, the Kaiser-Meyer-Olkin (KMO) and Bartlett's test of sphericity values both exceeded the desired thresholds of greater than 0.6 and less than 0.05, respectively. KMO predicted the data would factor well while the Bartlett's test indicated acceptable correlation between variables. Five factors with eigenvalues greater than 1.0 were extracted accounting for 72.8% of the total variance. All items loaded according to hypothesized groupings and exhibited relatively high and close within each factor. That is, with the exception of CBEH (Championing Behaviors). The large spread between the maximum and minimum loadings was cause for concern. However, the construct proved to exhibit both discriminant and convergent validity (confirmed in a following section) and so, the four items were retained.

The CMV factor loadings were statistically significant indicating that common method variance would have biased the results had we not controlled for it. All goodness of fit statistics presented in this study were calculated from models that controlled for CMV. It was not only model fit indices which accounted for CMV, but also the regression weights in the structural model.

After controlling for common method variance, the reliability and validity of each construct were assessed utilizing standardized factor loadings, composite reliabilities (CR), average variance extracted (AVE), maximum shared variance (MSV), and average shared variance (ASV) (Fornell and Larcker, 1981; Hair et al., 2006). The criteria for convergent validity includes standardized factor loadings >0.50, AVE > 0.50, and *CR* > 0.70. Per Table 4, all variables and constructs exceed desired thresholds except for the AVE of the Championing Behaviors construct and the standardized coefficient of one of its items, CBEH3. However, because the EFA inferred potential issues with different CBEH variables and based on the strength of other statistics, particularly Cronbach's alpha and composite reliability, no changes were made. Each construct also met the criteria for internal reliability with a Cronbach's alpha and composite reliability exceeding 0.70 (Fornell and Larcker, 1981; Nunnally and Bernstein, 1994).

**Table 4:** **Convergent and discriminant validity statistics**.

**Constructs/Dimensions**	**Coefficients^a^**	***T*-value**	**Cronbach's Alpha**	***CR*^b^**	**AVE^c^**	**MSV^d^**	**ASV^e^**
Autonomous motivation (AMOT)			0.80	0.74	0.50	0.42	0.25
AREG1	0.544	7.500					
AREG3	0.818	13.311					
AREG6	0.728	11.284					
Organizational support (OSUP)			0.87	0.82	0.61	0.20	0.11
OSUP1	0.613	9.209					
OSUP4r	0.848	13.832					
OSUP5	0.853	14.898					
Shared vision (SVIS)			0.93	0.89	0.74	0.31	0.20
SVIS1	0.909	17.484					
SVIS2	0.896	17.217					
SVIS5	0.758	13.161					
In-role behaviors (IBEH)			0.93	0.87	0.70	0.09	0.04
IBEH1	0.855	14.461					
IBEH3	0.762	11.650					
IBEH5	0.880	15.102					
Championing behaviors (CBEH)			0.86	0.78	0.48	0.42	0.22
CBEH1	0.848	13.456					
CBEH2	0.785	12.441					
CBEH3	0.436	5.505					
CBEH4	0.638	9.260					

a *Standardized factor loadings*.

b *Composite Reliability*.

c *Average Variance Extracted*.

d Maximium Shared Variance

e *Average Shared Variance*.

AVE varies from 0 to 1, and it represents the ratio of the total variance that is due to the latent variable. Using the logic as presented earlier, an AVE of 0.5 or more indicates satisfactory convergent validity, as it means that the latent construct accounts for 50% or more of the variance in the observed variables, on the average. If AVE is less than 0.5, the variance due to measurement error is larger than the variance captured by the construct, and the validity of the individual indicators, as well as the construct, is questionable. Note that AVE is a more conservative measure than CR. On the basis of CR alone, the researcher may conclude that the convergent validity of the construct is adequate, even though more than 50% of the variance is due to error. One should also interpret the standardized parameter estimates to ensure that they are meaningful and in accordance with theory (Malhotra and Dash, 2011, p. 702).

To evaluate discriminant validity, AVE for each construct must be >0.50 and exceed the values of MSV and ASV (Fornell and Larcker, 1981). Again, all constructs exceed desired thresholds except for Championing Behaviors. Because its AVE is greater than its MSV or ASV and based on the strengths of other statistics, the Championing Behaviors construct was considered to have acceptable discriminant validity.

The hypothesized structural model was developed and evaluated in AMOS. It should be noted that all structural model statistics were calculated after inclusion of the control variables acquisition age (AAGE), participant tenure (PTEN, and participant gender PGEN). Table 5 contains the resulting goodness of fit statistics. The initial model approached good fit, however, the modification indices suggested that the addition of three regression paths would improve model fit. Paths from Individual Performance Incentives to Championing Behaviors, Organizational Support to In-Role Behaviors, and Shared Vision to In-Role Behaviors were added to the model and analyzed. While the fit did not improve much, all added paths were statistically significant and therefore, were retained in the final structural model. The respecified model and impact of these non-hypothesized paths will be discussed at length in the Findings and Discussion sections.

**Table 5:** **SEM goodness of fit statistics for the structural model**.

**Goodness of fit**	**Criteria for good fit**	**Initial structural model**	**Respecified structural model**
**FIT OF PREDICTED VS. OBSERVED COVARIANCES**			
Chi-square (df)	N/A	391.7 (151)	363.1 (148)
Relative chi-square (CMIN/DF)	<3.0^a^	2.6	2.1
SRMR	≤0.08^b^	0.06	0.05
**FIT OF DEFAULT VS. INDEPENDENCE MODELS**			
CFI	≥0.95^b^	0.93	0.94
NFI	≥0.90^c^	0.90	0.91
TLI	≥0.95^b^	0.91	0.92
**FIT OF PREDICTED VS. OBSERVED COVARIANCES BUT**			
RMSEA (90% CI)	≤0.06^b^	0.075 (0.066–0.084)	0.072 (0.062–0.081)

a *Kline (1998)*.

b *Hu and Bentler (1999)*.

c *Bentler and Bonett (1980)*.

**Figure 3:**
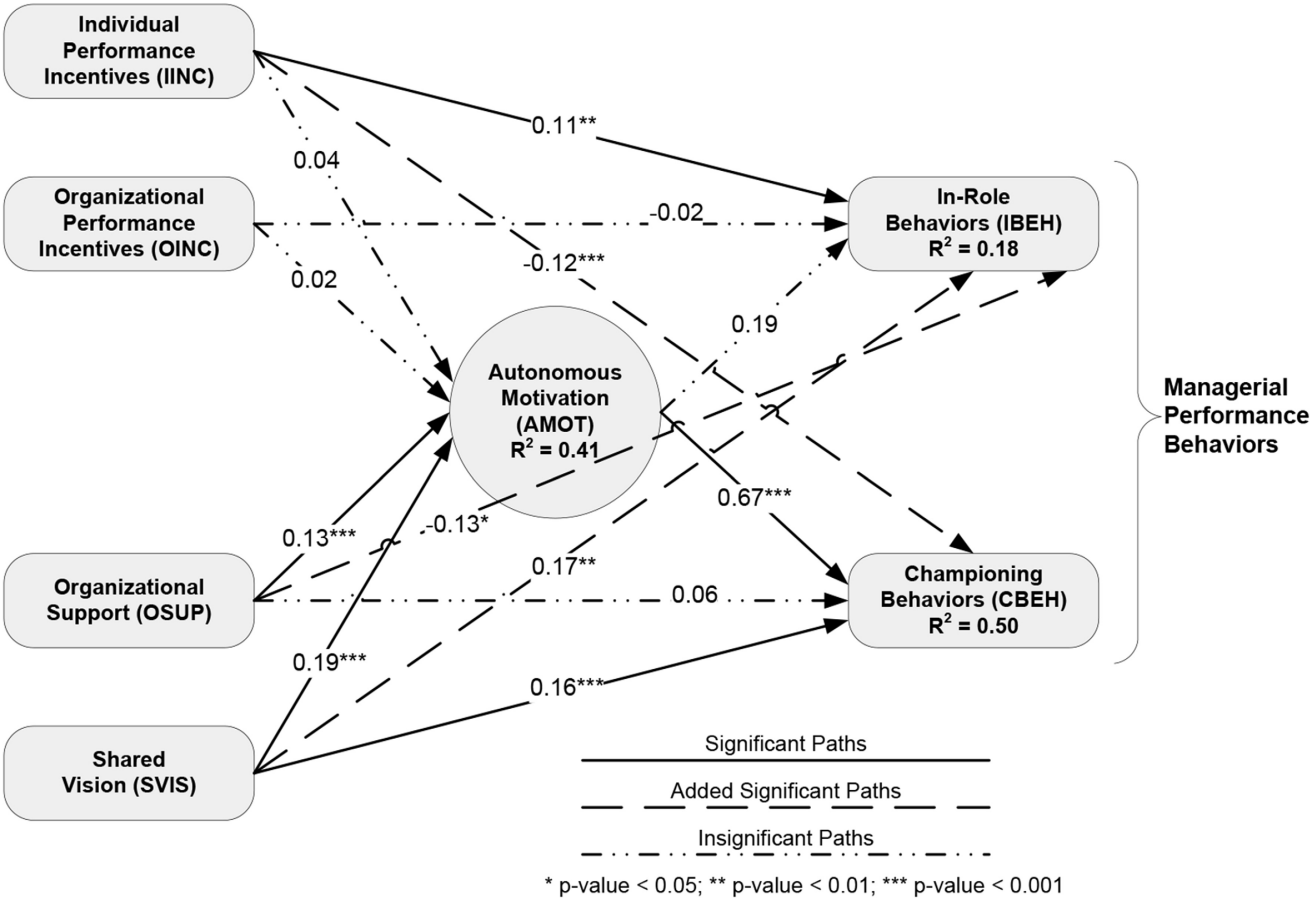
**Path diagram**.

Table 6 lists the statistical relationships between the constructs in the final structural model. The *r*^2^ statistic represents the amount of variability in the dependent variable that can be explained by the independent variables. The unstandardized coefficient indicates raw strength of the influence of each independent variable on the dependent variable. Finally, the *t*-value provides the significance of each coefficient. A graphical representation of the structural model summarizing the statistical relationships between constructs is shown in Figure 3.

**Table 6:** **Statistical relationships between structural model constructs**.

**Dependent variable**	**R-square**	**Independent variables**	**Unstandardized coefficient**	***T*-value**	**Standard error**
Autonomous motivation (AMOT)	0.41	Individual performance incentives (IINC)	0.038	1.62	0.024
		Organizational performance incentives (OINC)	0.019	1.36	0.014
		Organizational support (OSUP)	0.134	3.45	0.039
		Shared vision (SVIS)	0.190	4.47	0.042
In-role behaviors (IBEH)	0.18	Individual performance incentives (I-INC)	0.107	2.70	0.039
		Organizational performance incentives (OINC)	−0.021	−0.85	0.024
		Shared vision (S-VIS)	0.169	2.78	0.061
		Organizational support (OSUP)	−0.131	−2.37	0.055
		Autonomous motivation (AMOT)	0.185	1.19	0.155
Championing behaviors (CBEH)	0.50	Individual performance incentives (I-INC)	−0.115	−3.90	0.029
		Organizational support (OSUP)	0.064	1.55	0.041
		Shared vision (SVIS)	0.158	3.57	0.044
		Autonomous motivation (AMOT)	0.672	5.06	0.133

The researchers evaluated the mediating effects of Autonomous Motivation using techniques developed by Mathieu and Taylor (2006). These techniques constrain each path in a mediated relationship in an iterative process to determine their significance using methods such as the Sobel test and bootstrapping (Mathieu and Taylor, 2006). Mathieu and Taylor (2006) assert that these techniques will identify indirect effects that other methods will reject. Indirect effects describe variables that mediate the relationship between independent and dependent variables that are not significantly correlated to each other. In other words, the absence of significant total effect between independent and dependent variables often cause researchers to mistakenly reject significant mediated relationships. Table 7 lists a summary of the results of mediation testing.

**Table 7:** **Mediation effects**.

**Hypothesized mediated paths**	**Mediation effect of autonomous motivation (A-MOT)**
Individual incentives (I-INC) ⇒ In-role behaviors (I-BEH)	No mediation; direct effect
Individual incentives (I-INC) ⇒ Championing behaviors (C-BEH)	Partial mediation
Organizational incentives (O-INC) ⇒ In-role behaviors (I-BEH)	No mediation
Organizational incentives (O-INC) ⇒ Championing behaviors (C-BEH)	No mediation
Organizational support (O-SUP) ⇒ In-role behaviors (I-BEH)	No mediation; direct effect
Organizational support (O-SUP) ⇒ Championing behaviors (C-BEH)	Indirect effects mediation
Shared vision (S-VIS) ⇒ In-role behaviors (I-BEH)	No mediation
Shared vision (S-VIS) ⇒ Championing behaviors (C-BEH)	Partial mediation

A multigroup analysis (Jöreskog, 1971; Byrne, 2001) assessed the moderating effects of senior vs. middle managers and managers under 45 years old vs. those over 45. The analysis for each group was conducted in three steps. First, the factor loadings of the AMOS measurement model were constrained equal and compared to the unconstrained model. A significant difference between each model's chi-square at a 90% confidence interval served as the threshold to reject the null hypothesis that the measurement model was invariant across groups. Because the measurement model was not invariant across management level or age groups, each constraint was released and compared via subsequent models to determine which specific factors were non-invariant. Finally, the non-invariant factors were allowed to estimate freely, which raised the chi-square significance to 0.183 for senior vs. middle managers and 0.083 for managers under vs. over 45 years old.

A similar procedure was used to assess structural model invariance. The causal paths between constructs were constrained equal and compared across groups. Interestingly, all paths were invariant across management level groups while none of the paths were invariant across the manager age groups. Releasing each of the constraints across age groups revealed specific differences of how our hypothesized model applied to managers over and under 45 years old. Table 8 provides information useful in deciphering and discussing these differences in the next sections.

**Table 8:** **Results of multigroup analysis for managers over and under 45 years old**.

**Construct paths**	**Under 45**		**45 and over**	
	**Coefficient**	***T*-value**	**Coefficient**	***T*-value**
Individual incentives (IINC) ⇒ In-role behaviors (IBEH)	0.132	2.078	0.114	1.058
Individual incentives (IINC) ⇒ Championing behaviors (CBEH)	−0.278	−4.278	−0.185	−2.006
Individual incentives (IINC) ⇒ Autonomous motivation (AMOT)	0.020	0.501	0.173	2.846
Organizational incentives (O-INC) ⇒ In-role behaviors (I-BEH)	−0.179	−2.627	−0.033	−0.298
Organizational incentives (OINC) ⇒ Autonomous motivation (AMOT)	0.133	3.115	−0.130	−2.295
Organizational support (O-SUP) ⇒ In-role behaviors (I-BEH)	−0.202	−2.910	0.196	1.673
Organizational support (O-SUP) ⇒ Championing behaviors (C-BEH)	0.259	3.575	0.137	1.285
Organizational support (O-SUP) ⇒ Autonomous motivation (AMOT)	0.125	2.887	0.233	3.177
Shared vision (S-VIS) ⇒ In-role behaviors (I-BEH)	0.146	1.931	0.388	2.975
Shared vision (S-VIS) ⇒ Championing behaviors (C-BEH)	0.283	3.465	0.254	2.633
Shared vision (S-VIS) ⇒ Autonomous motivation (AMOT)	0.262	5.307	0.226	2.965
Autonomous motivation (AMOT) ⇒ In-role behaviors (I-BEH)	0.570	4.023	−0.152	−0.465
Autonomous motivation (AMOT) ⇒ Championing behaviors (C-BEH)	0.346	2.486	0.151	0.494

*Coefficients listed are unstandardized regression weights*.

The structural model also controlled for acquisition age, manager tenure and manager gender. Of nine possible effects (nine paths connecting three controls to two dependent and one mediating variables), only two were significant. Managers' tenure was likely to positively influence their in-role behaviors (unstandardized regression coefficient = 0.155; *t*-value 2.77) and acquisition age was likely to positively influence managers' autonomous motivation (unstandardized regression coefficient = 0.125; *t*-value 2.26).

## Discussion

Results of the hypotheses tested are shown in Table 9. For a listing of *r*^2^ values, regression coefficients and *t*-values please refer to Table 6 and Figure 3.

**Table 9:** **Summary of hypothesis testing**.

**Hypothesis**	**Finding**
Hypothesis 1: Individual performance incentives positively influence in-role behaviors after controlling for autonomous motivation	Supported
Hypothesis 2: Organizational performance incentives positively influence in-role behaviors after controlling for autonomous motivation	Rejected
Hypothesis 3: Individual performance incentives have a stronger (more positive) influence on in-role behaviors than organizational performance incentives	Supported
Hypothesis 4: Individual performance incentives negatively influence autonomous motivation	Rejected
Hypothesis 5: Organizational performance incentives negatively influence autonomous motivation.	Rejected
Hypothesis 6: Autonomous motivation positively influences in-role behaviors.	Rejected
Hypothesis 7: Autonomous motivation positively influences championing behaviors	Supported
Hypothesis 8: Autonomous motivation has a stronger (more positive) influence on championing behaviors than in-role behaviors	Supported
Hypothesis 9: Autonomous motivation negatively mediates the influence of individual performance incentives on in-role behaviors	Rejected
Hypothesis 10: Autonomous motivation negatively mediates the influence of organizational performance incentives on in-role behaviors	Rejected
Hypothesis 11: Autonomous motivation negatively mediates the influence of individual performance incentives on championing behaviors	Rejected
Hypothesis 12: Autonomous motivation negatively mediates the influence of organizational performance incentives on championing behaviors	Rejected
Hypothesis 13: Organizational support positively influences autonomous motivation	Supported
Hypothesis 14: Organizational support positively influences championing behaviors	Rejected
Hypothesis 15: Autonomous motivation positively mediates the influence of organizational support on in-role behaviors	Rejected
Hypothesis 16: Autonomous motivation positively mediates the influence of organizational support on championing behaviors	Supported
Hypothesis 17: Share vision positively influences autonomous motivation	Supported
Hypothesis 18: Share vision positively influences championing behaviors	Supported
Hypothesis 19: Autonomous motivation positively mediates the influence of shared vision on in-role behaviors	Rejected
Hypothesis 20: Autonomous motivation positively mediates the influence of shared vision on championing behaviors	Supported

### Influences on championing behaviors

The combination of performance incentives, organizational support, shared vision and autonomous motivation accounted for 50% of the variance in championing behaviors. Shared vision had a direct, positive and highly likely influence on championing behaviors. In fact, it was the only independent variable to have a direct positive impact on championing behaviors.

Shared vision also indirectly influenced championing behaviors via autonomous motivation, indicating partial mediation. The total effect of shared vision on championing behaviors was 0.286. Comparing the total effects of the independent variables, shared vision had a stronger impact on championing behaviors than individual incentives, organizational incentives and organizational support combined.

An unexpected finding indicated that individual performance incentives directly reduced championing behaviors. At first glance, this was counterintuitive because individual incentives target in-role behaviors only. However, researchers have proposed an inverse relationship between in-role and organizational citizenship behaviors (OCBs). Bergeron (2007) proposed that under an outcome based reward system, increases in OCBs will result in decreases in in-role behaviors and vice-versa. Given the fact that OCBs and championing behaviors are both forms of extra-role behaviors (Morrison and Phelps, 1999), it appears that our findings reflect Bergeron's work.

The mediation analysis reported a significant and positive indirect mediation between organizational support and championing behaviors via autonomous motivation. In this case, the indirect mediation at nearly 50% stronger than the non-significant direct effect, 0.09 vs. 0.06, respectively.

The mediation analysis also showed that autonomous motivation partially mediated the influence of individual incentives on championing behaviors. This finding conflicted with the unconstrained analysis in Figure 3 depicting the path between individual incentives and autonomous motivation as non-significant (negating mediation). As such, the mediation analysis provided the more accurate assessment of the mediation relationship by isolating individual paths to better examine their effects and by using bias-corrected bootstrapping for estimation.

The resulting finding indicated that autonomous motivation positively mediated the influence of individual incentives on championing behaviors. However, the unexpected negative direct effect between individual incentives and championing behaviors resulted in a negative total effect. It should also be noted that the positive indirect effect of individual incentives on championing behaviors was very weak. The regression coefficient of 0.03 was only 25% of the direct effect.

Finally, organizational support did not positively influence championing behaviors. In addition to a low *t*-value of 1.55, organizational support had a weak influence on championing behaviors with a regression coefficient of 0.06.

### Influences on in-role behaviors

The combination of performance incentives, organizational support, shared vision and autonomous motivation accounted for only 18% of the variance in in-role behaviors. Of the three hypothesized direct effects, only one, individual incentives had a significant effect. Neither organizational incentives nor autonomous motivation significantly influenced in-role behaviors. Because autonomous motivation did not significantly influence in-role behaviors, none of the mediation hypotheses for in-role behaviors were significant. However, we caution the confidence in this finding because of the high standard error of 0.155 for the autonomous motivation (AMOT) → in-role behaviors (IBEH) path which certainly increases the chance for a Type 1 error. A lower standard error would negate the rejection of several of the mediation hypotheses, substantially changing the findings of the overall study. In consideration of subsequent research, researcher should improve the items measuring autonomous motivation to achieve more accurate predictions of mediation hypotheses.

The most interesting finding here was how little organizational incentives influenced in-role behaviors. With a direct effect of −0.021, indirect effect of 0.004 and total effect of −0.017, all values were non-significant. This finding suggested that the common practice of providing substantial organizational incentives to M&A managers is totally ineffective.

An unexpected finding revealed that shared vision directly and positively influenced in-role behaviors. In fact, the influence of shared vision on in-role behaviors was 35% stronger than the influence of individual incentives on in-role behaviors with regression coefficients of 0.17 and 0.11, respectively. This is quite intriguing considering the strong pay-performance link between individual incentives and in-role behaviors asserted in compensation literature (Bucklin and Dickinson, 2001; McGee et al., 2006). We believe this may be an engaging topic for further research.

The previous section discussed the opposite effects that individual incentives had on in-role and championing behaviors and how it supported the work of Bergeron (2007). In other words, these opposite effects mimicked the inverse relationship between in-role and OCBs proposed by Bergeron. A similar phenomenon was caused by the negative direct effect of organizational support on in-role behaviors vs. the positive indirect effect of organizational support on championing behaviors. As organizational support increased, championing behaviors increased and in-role behaviors decreased.

### Influences on autonomous motivation

The combination of performance incentives, organizational support, and shared vision accounted for 41% of the variance in autonomous motivation. Organizational support and shared vision were the major predictors of autonomous motivation virtually contributing all of the effect. Conversely, the regression coefficients linking individual and organizational incentives to autonomous motivation were among the weakest in the study at 0.04 and 0.02, respectively. This is an interesting outcome considering SDT posits that incentives decrease autonomous motivation. In this study and setting, SDT did not hold. In fact, the effects of performance incentives on autonomous motivation paled in comparison to the effects of organizational support and shared vision on autonomous motivation.

### Comparative hypotheses

Hypotheses 3 and 8 predicted the comparative strengths of two pairs of conceptualized paths. Hypothesis 3 asserted that the stronger pay-performance link between individual incentives and in-role behaviors would result in a stronger influence than organizational incentives. The influence of individual incentives was not only substantially stronger but was significant as compared to a weak and non-significant effect for organizational incentives.

Hypothesis 8 posited that autonomous motivation would be a stronger predictor of championing behaviors than in-role behaviors. The data supported this hypothesis. The regression coefficient for its effect on championing behaviors was 3.5 times stronger than its effect on in-role behaviors. In addition, the data suggested much more confidence in the ability of autonomous motivation to predict championing behaviors with a *t*-value of 5.06 compared to 1.19.

### Controls

The multigroup SEM analysis indicated that the structural model was invariant across senior and middle managers but not invariant across managers over and under 45 years old. Table 8 lists the regression coefficients and *t*-values for each path of the diagram depicted in Figure 3 for managers over and under 45 years old.

Referring to Table 8, there are 13 paths that AMOS reported as significantly different for managers under vs. over 45 years old. Of the 13 paths, 8 of them disagreed on significance, that is, one path was significant and the other was not significant. Of those 8 paths, 6 of them have regression coefficients with differences >0.02. These 6 paths represent the major differences in construct relationships between managers under 45 and those over 45 and are listed in Table 10.

**Table 10:** **Construct paths w/opposite significance and regression differences >0.02**.

**Construct paths**	**Under 45**		**45 and over**	
	**Coefficient**	***T*-value**	**Coefficient**	***T*-value**
Organizational support (O-SUP) ⇒ Championing behaviors (C-BEH)	0.259	3.575	0.137	1.285
Autonomous motivation (AMOT) ⇒ Championing behaviors (C-BEH)	0.346	2.486	0.151	0.494
Organizational incentives (O-INC) ⇒ In-role behaviors (I-BEH)	−0.179	−2.627	−0.033	−0.298
Shared vision (S-VIS) ⇒ In-role behaviors (I-BEH)	0.146	1.931	0.388	2.975
Autonomous motivation (AMOT) ⇒ In-role behaviors (I-BEH)	0.570	4.023	−0.152	−0.465
Individual incentives (IINC) ⇒ Autonomous motivation (AMOT)	0.020	0.501	0.173	2.846

*Coefficients listed are unstandardized regression weights*.

Per Table 10, the championing behaviors of managers under 45 were more sensitive to organizational support and autonomous motivation than those over 45. The regression coefficients for younger managers were roughly twice the strength of these effects on older managers. The in-role behaviors of younger managers were also more sensitive to organizational incentives and autonomous motivation, reporting regression coefficients between 3.8 and 5.4 times those for older managers. These findings indicated that younger managers cared more about how the organization perceives them. The behaviors of younger managers were also more influenced by how much they internalized the organization's goals (autonomous motivation). In general, the data indicated that younger managers were much more sensitive to the support, incentives and goals of the organization than older managers.

Conversely, older managers cared more about shared vision, defined in this study as their alignment with the overall direction and purpose of the organization. Shared vision positively influenced the in-role behaviors of older managers more than 2.5 times that of younger managers. As individuals age, they are less likely to look for a new job (Martin, 1979; Griffeth et al., 2000). In mergers and acquisitions, individuals who identify with the vision of the organization are also less likely to look for a new job and more likely to increase their performance (Haslam, 2001; Cartwright, 2005). These two notions suggest that older M&A employees should be more sensitive to shared vision and that increases in shared vision should reduce turnover intent and increase performance.

The multigroup analysis also indicated that individual incentives influenced the autonomous motivation of older managers much more than younger managers. However, because autonomous motivation did not significantly influence either in-role or championing behaviors of older managers, this finding doesn't matter. Regardless of how much individual incentives impact the autonomous motivation of older managers, it does not significantly influence their behaviors.

The structural model controlled for acquisition age, manager tenure and manager gender. Of the nine possible effects, only two effects were significant. First, the findings reported a positive relationship between manager tenure and in-role behaviors with a regression coefficient of 0.16 and *t*-value of 2.77. This outcome seems intuitive as one would expect managers to become more efficient in their formal, in-role behaviors over time.

The findings also reported a significant positive relationship between acquisition age and autonomous motivation with a regression coefficient of 0.13 and a *t*-value of 2.26. These effects were consistent with researchers who posit that it takes time for employees to reconcile their feelings of uncertainty regarding large-scale organizational change (Liu and Perrewé, 2005), resulting in a temporary reduction in work motivation, specifically in mergers and acquisitions (Seo and Hill, 2005).

### M&A practice is wrong on championing behaviors

The findings directly conflict with one of the most common and longstanding M&A assumptions that organizational performance incentives induce discretionary behaviors from acquired managers (Kaplan, 2000; Hitt et al., 2001; Larsson and Finkelstein, 2002). According to the data, organizational incentives did not significantly affect championing behaviors directly or indirectly. In fact, AMOS reported the total effect of organizational incentives on championing behaviors at a negligible 0.01.

These findings suggest that the ubiquitous use of stock options and profit sharing plans in M&A may be a waste of time and money. Seemingly, the implication to practice would be to eliminate organizational performance incentives. However, the practice of offering acquired manager's stock options and profit sharing has become an expected practice. These expectations may contribute to the loss of incentive power, possibly explaining the poor effects of these incentives on manager behaviors. In either case, should an acquirer fail to offer organizational incentives or offer substantially reduced versions, the effect on management morale and turnover could be devastating to the company. This dilemma suggests an agenda for future research, exploring the effects of various levels of organizational performance incentives on managerial morale, turnover and performance in an M&A context.

### M&A practice is wrong on shared vision

Shared vision was the only independent variable that positively influenced both championing and in-role behaviors. Surprisingly, shared vision impacted in-role behaviors more than individual performance incentives, despite its strong pay-performance link. Shared vision was also one of only two variables to significantly influence autonomous motivation.

One of the first duties of acquirers is to establish performance incentives of their newly acquired managers. In fact, stock incentives for CEOs and senior managers are usually established prior to the official transaction as part of the legal paperwork Details of profit sharing plans and individual performance bonuses soon follow.

The study findings imply that M&A practitioners should establish a higher priority on shared vision. Other researchers have identified shared vision as essential to the successful performance of merged and acquired organizations (Haspeslagh and Jemison, 1991; Sitkin and Pablo, 2005). Serial acquirers such as GlaxoSmithKline and Cisco Systems promote shared vision as an important part of their acquisition successes (DiGeorgio, 2001; Stahl and Mendenhall, 2005). Whereas, these sources base their arguments on qualitative findings, the present study contributes quantitative evidence to support the importance of shared vision, directly comparing their effects to those of performance incentives in an M&A context. Furthermore, the strength of the quantitative results suggests that M&A practitioners should prioritize shared vision above performance incentives.

### Individual incentives and prganizational support

Study findings indicated that individual incentives positively influenced in-role behaviors and negatively influenced championing behaviors. The absolute strengths of these bipolar effects were almost identical with direct effects of 0.11 and −0.12, respectively. This finding was consistent with the assertions of some practitioners and researchers that individual incentives can work “too well,” causing individuals to focus almost exclusively on in-role behaviors to the detriment of extra-role behaviors (Kohn and Thompson, 1993; Wright et al., 1993; Deckop et al., 1999; Hall and Murphy, 2003). Acquirers commonly address this issue by providing organizational incentives in addition to individual incentives to induce extra-role behaviors that foster cooperation and teamwork (FitzRoy and Kraft, 1995). Our findings supported the idea that individual incentives decrease championing behaviors but did not support the idea that introducing organizational incentives would offset the decrease.

The data did support the idea that increasing organizational support would offset the negative effects of individual incentives on championing behaviors. Organizational support positively influenced championing behaviors (indirect effect 0.09) but also negatively influenced in-role behaviors (direct effect −0.13). This supports the work of researchers who argue that focus on extra-role behaviors reduce the performance of in-role behaviors (Bergeron, 2007).

By highlighting the gains and costs of increasing individual incentives and organizational support, the study findings provide insights for cultivating desired managerial behaviors. For example, increasing organizational support for managers charged with large-scale change efforts should help foster the championing behaviors required to overcome resistance to aggressive growth and change. Conversely, increasing individual performance incentives should help cultivate the in-role behaviors of managers charged with sustaining day-to-day operational efficiencies, especially during periods of environmental disruptions typical in M&As.

### Age matters

The effect of organizational support on younger managers' championing behaviors was roughly 2 times stronger than older managers. The effect of organizational incentives on younger managers' in-role behaviors were over five times stronger than older managers. The degree that younger managers internalized current organizational goals (autonomous motivation) affected their championing and in-role behaviors 2–3.5 times more than older managers. In contrast, shared vision influenced the in-role behaviors of older managers 2.5 times more than younger managers.

These findings imply a difference in temporal focus for younger vs. older managers. The items of concern for younger managers, organizational support, incentives and current goals represent current actions, promises or objectives. The concern for older managers, shared vision, focuses more on what the company will become in the future. While plausible, there is little evidence in the scope of the present study to support this explanation. However, this may be an interesting topic for future research.

As mentioned earlier, the effect of organizational incentives on younger managers' in-role behaviors were over five times stronger than older managers. This statistic implies that older managers performed their formal duties more consistently than younger managers, despite fluctuations in organizational incentives. This implication was supported by the substantially lower standard deviation for in-role behaviors of older vs. younger managers at 0.62 vs. 0.77. In addition, the mean for in-role behaviors of older managers (3.85) was higher than younger managers (3.72), inferring that older managers performed their formal duties better than younger managers as well as more consistently. The data suggests that acquirers should note the ages of their managers when designing organizational incentive plans.

### Limitations

The researchers acknowledge several limitations of this study. First, the study is cross-sectional which means the causal relationships can only be hypothesized from previous research and theory. Future research should utilize a longitudinal approach as a more rigorous analysis of our proposed causal relationships.

Second, common method variance was present due to the nature of the self-report data. Therefore, we modeled a latent common method factor that was constrained to load equally on all observed variables in the measurement and structural models. By doing so, we attempted to partial out the variance due to the common method.

Finally, the sample included M&As owned by private equity firms only. This is but one segment of many in the M&A domain. Replication of the study exploring other segments would be required to test the generalizability of our findings.

## Conclusion

To our knowledge, this is the first M&A study to integrate financial and psychological drivers of managerial performance into a single testable model. Although this initial study certainly requires further testing and refinement, we assert that the findings provide valuable insights toward understanding the drivers of managerial behaviors within mergers and acquisitions. Specifically, the study provides evidence that shared vision is far more effective at driving managerial performance, as defined by in-role and championing behaviors, than common M&A practices of providing financial incentives. This is an important step forward in reducing the dismal failure rates that continue to plague the M&A domain.

### Conflict of interest statement

The author declares that the research was conducted in the absence of any commercial or financial relationships that could be construed as a potential conflict of interest.

## Footnotes

^1^Acquisition premium: The actual cost of acquiring a target versus its book value, market value, or estimated value. Sirower (2000) estimates that the average acquisition premium is in excess of 40%.
